# INTERVENTIONS FOR SENSE OF COHERENCE AMONG OLDER ADULTS WITH CHRONIC DISEASE: A SYSTEMATIC REVIEW AND META-ANALYSIS

**DOI:** 10.1093/geroni/igad104.2605

**Published:** 2023-12-21

**Authors:** Yaqian Liu, Angela Y M Leung, Jed Montayre

**Affiliations:** The Hong Kong Polytechnic University, Hong Kong, Hong Kong; The Hong Kong Polytechnic University, Hong Kong, Hong Kong; The Hong Kong Polytechnic University, Hong Kong, Hong Kong

## Abstract

**Background:**

Sense of Coherence (SoC) is a core concept of “salutogenesis” in positive psychology, correlated with emotional distress and disease development in older adults with chronic disease (OACD). A diversity of interventions has been developed to enhance SOC, but research findings are conflicting and only a few studies have reviewed the effectiveness. Objective: This paper aimed to explore appropriate interventions and evaluate the effectiveness of these SoC interventions on OACD.

**Methods:**

Search terms including ‘sense of coherence’, ‘older adult’ and ‘randomized controlled trial’ were performed in nine electronic databases and two grey literature sources. Publications were written in English from January 1979 to January 2023. A narrative synthesis was performed to determine intervention details, and classical meta-analysis was used to analyze available data on SoC using RevMan.

**Results:**

27 RCT studies were identified, involving salutogenic-based program, cognitive behavior treatment, and self-management intervention. Meta-analysis was carried out with 21 RCTs involving 1,687 patients. It showed significant effects of all interventions on SoC, compared to usual care, among OACD (SMD=0.19, 95%CI [0.09, 0.28], p<0.001) and at 3-month follow-up (SMD=0.14, 95%CI [0.03, 0.24], p<0.001), but no significant difference was observed at 6-month or 12-month follow-up (SMD=0.12, 95%CI [-0.13, 0.43], p=0.07; SMD=0.15, 95%CI [-0.27, 0.47], p=0.09).

**Conclusions:**

Interventions for SoC were shown to be effective right after the intervention and at 3 months. However, its effects were not sustained over a longer period. Further research is needed to explore optimal duration and components of interventions for SoC in this population.

